# Session-RPE for quantifying workload in olympic curling athletes

**DOI:** 10.3389/fspor.2025.1636827

**Published:** 2025-09-10

**Authors:** Junqi Wu, Chunlei Li

**Affiliations:** Academy of Strength Training and Conditioning, Beijing Sport University, Beijing, China

**Keywords:** load monitoring, sRPE, Olympic, curling, omegawave

## Abstract

**Objective:**

To investigate the correlation between different workload methods among Olympic curling athletes.

**Materials and methods:**

Eight curlers were monitored after training during Olympic seasons with three load quantification methods: external load measurements, physiological/biochemical markers, and Omegawave state indices. Intraclass Correlation Coefficient and Bland-Altman plots were used to analyze the Session-RPE index [sRPE workload (RPE × session duration), acute:chronic workload ratio (ACWR), etc.], external [number of draws (the number of curling stones thrown during training/competition), training duration, etc.], and internal [physiological and biochemical indices (testosterone, etc.), and Omegawave sport performance evaluation system indices (comprehensive readiness, etc.)] workloads.

**Results:**

The sRPE index was significantly correlated with external loads and Omegawave sport performance indicators at the 0.01 level (*p* < 0.01); it was significantly correlated with cortisol and creatine kinase at the 0.05 level (*p* < 0.05). In the standardized ICC and Bland-Altman plot concordance analyses, the sRPE correlates showed moderate (0.4 < ICC < 0.6) to strong (0.6 < ICC < 0.8) concordance with the corresponding external loading indices, the Omegawave athletic status indices, and average (0.2 < ICC < 0.4) to moderate agreement with the corresponding physiological and biochemical indicators.

**Conclusions:**

The sRPE is a valid curling training-load tool capturing sport-specific demands but retains psychosocial limitations. Appropriate methods should be selected based on actual conditions and needs when choosing how to quantify and evaluate training load.

## Introduction

Curling, a strategically guided team sport, exhibits distinct characteristics including prolonged duration, intermittent high-intensity efforts, and significant cognitive demands (1, 2). These attributes necessitate specialized load monitoring approaches. Given that tactical decisions substantially modulate in-game load magnitudes, discrete quantification methods (e.g., single-stone presses or sweep frequency) prove inadequate for curling load evaluation. Such metrics become temporally diluted over 2–3 h matches, potentially yielding reductive assessments when used exclusively. Among quantitative load-monitoring tools applied in team sports (e.g., football, basketball, volleyball), sRPE offers superior cost-effectiveness, portability, universality, timeliness, accuracy, and non-invasiveness. Crucially, sRPE uniquely accounts for athletes' psychological exertion during training and competition, but the application of sRPE or comparable load monitoring tools remains underdeveloped among curling athletes.

**Table 1:** Basic information table of experimental subjects.

Information	All (*n* = 8)
Age (year), mean (range)	26.8 (22–31)
Height (cm), mean (SD)	181.1 (4.9)
Weight (kg), mean (SD)	77.2 (5.5)
BMI (kg/m^2^), mean (SD)	23.5 (1.3)
Skeletal muscle (kg), mean (SD)	38.6 (3.0)
Body fat (%), mean (SD)	12.6 (1.6)
Training year (year), mean (SD)	8.6 (2.4)

**Table 2:** General description of the data.

Mean ± SD	Position 1st & 2nd	Position 3rd & 4th	All
Acute load	7,063 ± 1,865.51	6,583 ± 1,664.31	6,804 ± 1,772.04
Chronic load	6,945 ± 1,073.55	6,513 ± 1,173.57	6,712 ± 1,146.44
ACWR	1.02 ± 0.23	1.01 ± 0.19	1.02 ± 0.21
Monotony	4 ± 1.48	5 ± 0.76	4 ± 1.20
Training pressure	27,741 ± 10,258.38	30,817 ± 7,924.13	29,399 ± 9,182.41
Curling load	6,179 ± 1,827.91	5,701 ± 1,570.13	5,921 ± 1,706.62
Curling duration	848 ± 245.71	866 ± 239.29	858 ± 241.89
Total duration	986 ± 248.26	1,011 ± 253.18	999 ± 250.66
Training draws	156 ± 76.08	164.53 ± 78.59	161 ± 77.37
Competition draws	78 ± 50.61	72 ± 49.67	75 ± 50.07
Total draws	234 ± 67.17	237 ± 68.07	236 ± 67.52

Curling Load: Training load accumulated by athletes during curling-specific training; Curling Duration: Time duration expended by athletes in curling-specific training sessions.

Borg pioneered the Rating of Perceived Exertion (RPE) in the 1960s–1970s to quantify physical exertion perception, subsequently developing the 6–20, CR-10, and CR-100 scales (3). Banister advanced this field by proposing the stimulus-fatigue model and Training Impulse (TRIMP) metric, enabling heart rate-based quantification of internal load across sports (4). Building on this work, Foster optimized the CR-10 scale (now the dominant RPE instrument in competitive sports) and introduced the Session-RPE (sRPE) method (5). This technique quantifies training/competition load by multiplying session duration (min) by post-session RPE, expressed in arbitrary units (a.u.) (5). As a practical metric of average intensity, sRPE enables effective exercise load quantification (6).

sRPE demonstrates strong correlations with physiological markers including heart rate (7), blood pressure (8), blood lactate, cortisol (9), and lactate threshold (10). Researchers have further utilized the RPE/blood lactate ratio for load analysis (11). While sRPE shows limited utility for resistance training evaluation (12), it correlates with Repetition in Reserve (RIR) metrics (13). Resistance training modalities differentially affect RPE scores, with high-intensity/low-repetition protocols yielding higher values than low-intensity/high-repetition regimens (14). sRPE associations extend to: 1. Total external workload (frequency × load) (15); 2. Training duration (16); 3. Equivalent training volume with varied loading patterns (17–20); 4. Work:rest ratios (21).

Sport-specific correlations exist with program parameters [e.g., jump count (22), IMA metrics (acceleration, deceleration, direction changes) (23)], though no relationships emerge with instantaneous power, contraction time, or jump height (24). In mixed training, sRPE exhibits stronger heart rate correlations than TRIMP, while associating significantly with total and high-speed movement distances (17, 25). Technical-tactical applications (26) and positional demands (27) induce sRPE variability, potentially reflecting differential functional exertion. For instance, dance studies employ sRPE to quantify technical movement difficulty (28). Modulating factors include: 1. Ambient temperature extremes (29); 2. Psychological/environmental variables (e.g., affective states, social context, coach-athlete assessment disparities) (30–32); 3. Exogenous substances (e.g., caffeine) (33). sRPE evaluates athletes': 1. Training awareness (34); 2. Movement perception proficiency (35); 3. RPE reliability influenced by training experience (36). Coach-mediated CR-10 scale interpretations further impact RPE validity (37). Pedersen additionally introduced perceived exertion for discomfort (RFD), session displeasure/pleasure (SPDF), and exercise enjoyment (EES) as load intensity metrics (20).

Athletes exhibit minimal injury risk when the Acute:Chronic Workload Ratio (ACWR) ranges between 0.8 and 1.3, while ACWR > 1.5 significantly elevates injury incidence (38). Critiques of the rolling average method highlight its failure to account for decaying training adaptations and fatigue effects over time, suggesting acute loads warrant greater weighting. Consequently, Exponentially Weighted Moving Averages (EWMA) were implemented, demonstrating superior temporal load variation sensitivity vs. ACWR. Practically, reduced daily training load variability increases monotonicity, heightening overtraining risk (39). While some researchers employ meanPRE for overreaching assessment (40), others differentiate sRPE into breathlessness (sRPE-B), cognitive/technical (sRPE-T), lower-limb (sRPE-L), and upper-body (sRPE-U) components. Among these, sRPE-L correlates most strongly with overall RPE, followed by sRPE-B, sRPE-T, and sRPE-U (41). RPE serves both as an independent metric for training-group intensity (42) and cross-group recovery evaluation (43). Beyond Foster-Banister-Edward algorithms, advanced methodologies include: 1. Time-series modeling (EWMA, ARCH, GARCH) for load-injury analysis (38, 44, 45); 2. WER-modified TRIMP calculations addressing RPE's interval/intensity fluctuation limitations (46).

Operational focus centers on RPE reporting timing. Studies validate sRPE reliability at 10 (47), 15 (48), 20 (49), and 30 (50) min post-exercise, with Foster advocating 30-min assessments during coach-athlete interactions (6). Fixed-time collection is essential, as next-day recall introduces error (51). Consensus supports 15–30 min reporting windows to mitigate recency bias-preventing acute terminal high-intensity efforts from inflating perceived exertion beyond the session's mean load.

Scientific and systematic workload monitoring is critically important in curling. The sport places exceptionally high demands on athletes' physical conditioning, technical skills, and psychological resilience. Workload monitoring enables coaches to precisely quantify the stimulus imposed on athletes, ensuring training loads remain within the effective window for enhancing athletic capacity. This prevents undertraining or overtraining, optimizes training effects, and improves training efficiency. Workload monitoring provides coaches with objective data to analyze differences between athletes in various positions, facilitating the personalization of training plans to maximize each athlete's potential. Furthermore, monitoring competition loads helps characterize competition demands. Athletes can then replicate these demands in training to enhance their adaptability and stability during actual competition. This study aims to investigate the correlation between different workload monitoring methods among Olympic curling athletes. The hypothesis is that variations in session-RPE (sRPE) are synchronized with variations in other workload monitoring metrics.

## Materials and methods

### Subjects

The subjects were eight members of the Chinese National Men's Curling Training Team preparing for the Winter Olympic Games, with an average age of 26.8 years (22–31 years), an average height of 181.1 ± 10.7 years, an average body weight of 77.2 ± 5.5 years, and an average number of years of training of 8.6 ± 2.4 years (Table 1). All athletes completed an informed consent form. The collection period was 211 consecutive days.

The study implemented three load-monitoring modalities: external load metrics, physiological/biochemical markers, and Omegawave state indices. Omegawave and external load data were collected daily, while physiological/biochemical parameters were assessed at 15-day intervals, followed by time-synchronized analyses. This 15-day period constitutes a mesocycle within the preparation phase, comprising three 5-day microcycles. Each microcycle featured four training days followed by a rest day, as designed by Head Coach Lindholm Peja.

### Subjective fatigue index

The study quantified training load using three methods: external load measurements, physiological/biochemical markers, and Omegawave state indices. Athletes provided RPE via the Borg CR-10 scale 15–30 min post-training during field and physical sessions. Injured athletes without training were assigned 0 A.U. We also calculated training monotony, training strain, short-term (5-day) and long-term (20-day) loading, and the short-term:long-term load ratio (using a sliding-window average). Training duration (recorded to the nearest minute) was defined as the period from the start to end of formal training. The “start” denoted when athletes began coach-prescribed training after standardized warm-ups on the field. The “end” occurred when athletes completed prescribed tasks and exited the main training area, excluding post-session stretching/relaxation.

### Definition of the number of pots and training time

This study categorizes draws (curling stone throws) as either training draws or competition draws. Training draws encompass all stones thrown during practice, including coach-prescribed throws and athlete-initiated additional throws. Competition draws include those made during intra-squad scrimmages, simulated matches (where coaches directly set scenarios to mimic international opponents), and official matches. Drawing on training and match duration, we derived a secondary metric: curling density (draws per unit time). Higher density (more throws in less time) indicates reduced decision-making time and lower cognitive effort per throw, while lower density (fewer throws over longer duration) reflects greater time for tactical deliberation and higher cognitive effort. Thus, draw density serves as a proxy for the ratio of cognitive to physical effort. Specific draw types (e.g., guard, takeout) were not statistically analyzed, as their occurrence is heavily influenced by dynamic game tactics and strategy, limiting meaningful interpretation.

### Omegawave athletic state evaluation system

The Omegawave Athletic State Evaluation System, widely used in training practice for assessing athletes' immediate readiness (52), was employed to evaluate subjects 15–30 min after their final daily training session. This system qualitatively assessed central nervous system (CNS) status and cardiac function through simultaneous electroencephalogram (EEG) and electrocardiogram (ECG) analysis, including cardiac bioelectrical current activation levels. Primary evaluation indices comprised: Cardiopulmonary Regulation Functional State (1–7 points) and CNS Readiness State (1–7 points).

### Physiological and biochemical indicators

Physiological and biochemical markers were collected every 15 days under standardized protocols from the National Winter Sports Center of China to evaluate athlete fatigue. Testing occurred at 06:30 on the final rest day of each cycle, with athletes in a fasted state. Four indicators assessed physiological response to training load: blood urea, creatine kinase, testosterone, and cortisol. Testosterone was measured using Chemiluminescent Microparticle Immunoassay (CMIA) on an ARCHITECT i1000sr automated immunoassay analyzer (ARCHITECT i1000sr, Abbott Laboratories Co., Ltd, USA). Cortisol was determined by Enzyme-Linked Immunosorbent Assay (ELISA). Creatine Kinase (CK) activity was analyzed via the continuous monitoring (enzymatic kinetic) method using an OLYMPUS AU2700 analyzer (OLYMPUS AU2700, Olympus Corporation Co., Ltd, Japan). Hematocrit was assessed using the impedance method on an HT-ESR24 dynamic hematocrit analyzer (HT-ESR24, Zibo Hengtuo Analytical Instruments Co., Ltd, China). All analyses were performed by experienced technicians according to standardized protocols.

### Measurement procedures

**Figure 1:**
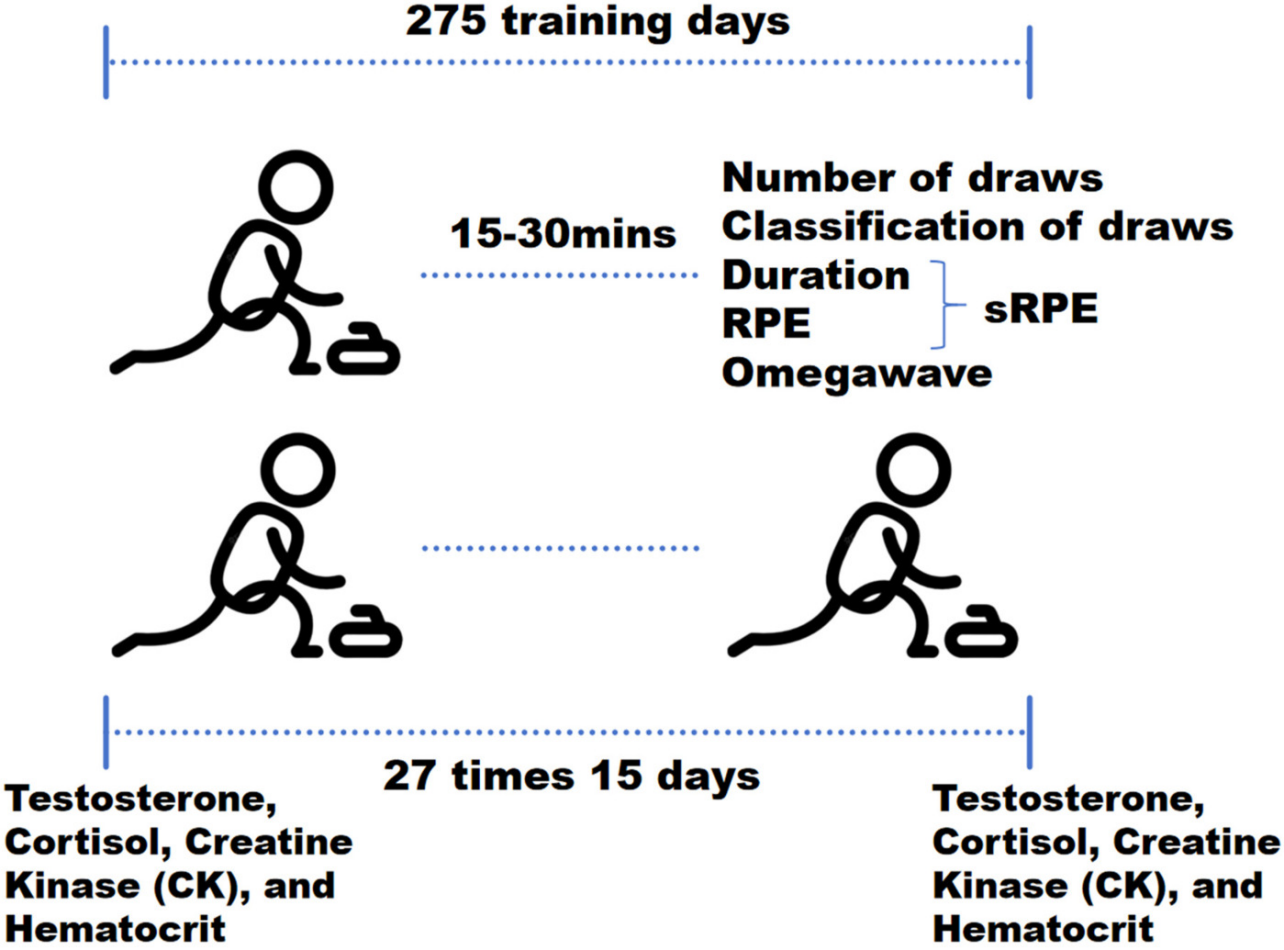
Measurement procedures.

During each training session, the number and type of stone deliveries were recorded for each athlete. Within 15–30 min post-training, session-RPE (sRPE) was collected. Athletes then performed Omegawave state assessments in isolated, quiet environments. On Day 15 of each cycle at 06:30 AM, fasting athletes provided samples for Testosterone, Cortisol, Creatine Kinase (CK), and Hematocrit assessment (Figure 1).

### Statistical methods

Data were processed using SPSS (version 26; SPSS, IBM Corporation, Armonk, New York, USA), WPS (version 2023; Kingsoft Office Software Co., Ltd., Beijing, China), and GraphPad Prism (version 9.5.1; GraphPad Software, Inc., San Diego, USA). Results are expressed as mean ± SD. All datasets met normality assumptions. Pearson correlation analyzed relationships between sRPE and: a. external load metrics, b. physiological/biochemical markers, and c. Omegawave Athletic Status indices. Spearman correlation was used for non-normally distributed data. Normality was assessed via Shapiro–Wilk test with *Q*–*Q* plots (*n* < 2,000) or Kolmogorov–Smirnov with *Q*–*Q* plots (*n* ≥ 2,000). Inter-metric consistency was evaluated using intraclass correlation coefficients (ICC) with Bland-Altman plots on standardized data (95% CI). ICC interpretation followed established thresholds: <0.20: Very poor; 0.21–0.40: Weak; 0.41–0.60: Moderate; 0.61–0.80: Substantial; 0.80: Excellent. Missing values were coded as 0. While this approach may introduce bias, the large sample size mitigates its impact. The computational formulas for sRPE, ACWR, Monotony, and Training Pressure addressed in this study (53) were:

Workload=RPE×trainingduration(min),A.U.(ArbitraryUnits)

$$\mathrm{ACWR}=\frac{\mathrm{Workloa}d_{1\mathrm{week}}}{\mathrm{Workloa}d_{\mathrm{Average}\;4\mathrm{weeks}}}$$

$$\mathrm{Monotony}=\frac{\mathrm{Workloa}d_{\mathrm{lastweek}}}{\mathrm{WeeklyWorkloa}d_{\mathrm{SD}}}$$

$$\mathrm{Training}\;\mathrm{Pressure}=\mathrm{Workloa}d_{\mathrm{week}}\times \mathrm{Monotony}$$

## Results

### General description of the data

This is the descriptive statistics of all data in this study (Table 2).

#### sRPE and external loads

**Figure 2:**
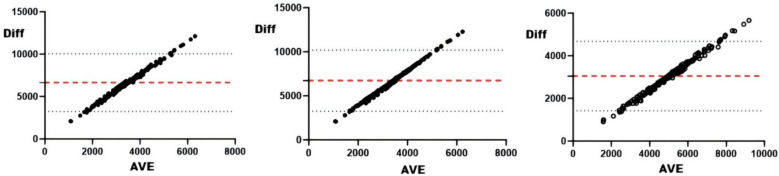
Bland-Altman plot of external loads and AL. From left to right: Bland-Altman plots of AL and training draws, competition draws, and total draws.

**Figure 3:**
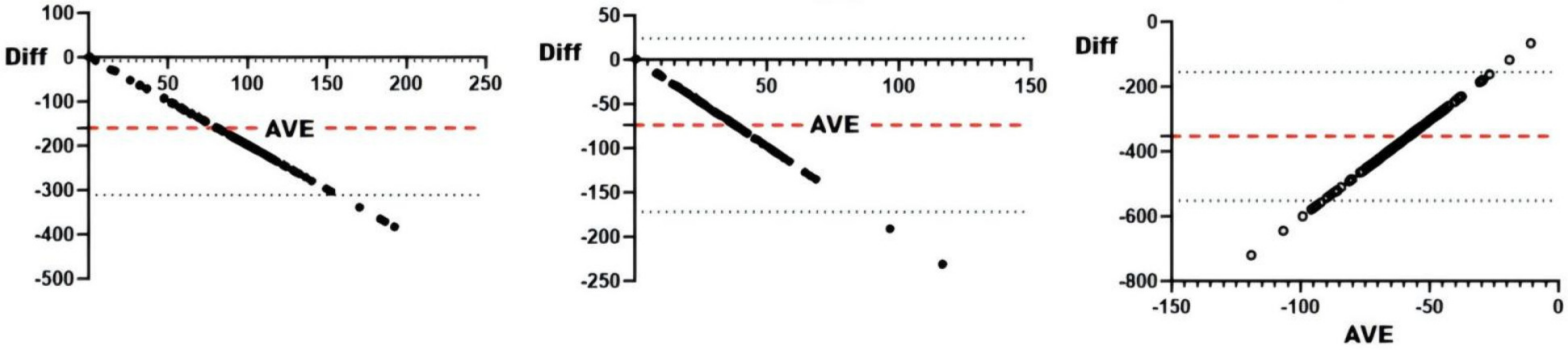
Bland-Altman plot of external loads and ACWRs. From left to right: Bland-Altman plots of ACWR and training draws, competition draws, and total draws.

**Figure 4:**
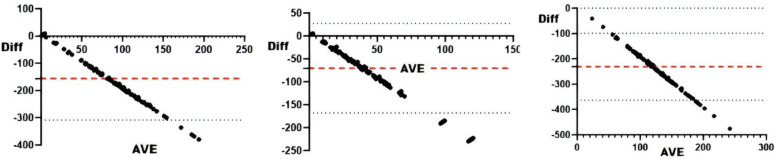
Bland-Altman plots of external load and monotonicity. From left to right: Bland-Altman plots of training monotony and training draws, competition draws, and total draws.

**Figure 5:**
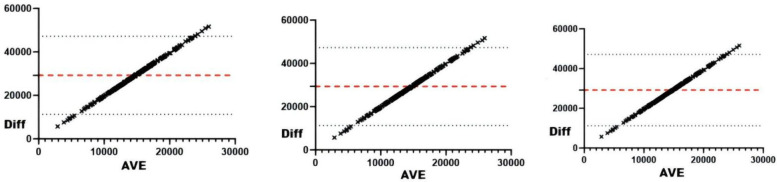
Bland-Altman plots of external load and training pressure. From left to right: Bland-Altman plots of training pressure and training draws, competition draws, and total draws.

Correlation analyses revealed significant associations (all *r* < .01) between sRPE and curling duration, total duration, training draws, and total draws (Table 3). The 5-day chronic load (CL) correlated significantly (*r* < .01) with time-based metrics (curling/total duration), volume metrics (training/total draws), and draw density, but showed no association with competition draws. The 5-day acute:chronic workload ratio (ACWR) demonstrated significant correlations (*r* < .01) with all duration and draw metrics. Training monotony correlated significantly with curling duration, total duration, and training draws (*r* < .01), but not competition or total draws, while training pressure showed moderate consistency with time-based metrics and total draws, and weak consistency with competition draws. Consistency analyses (Table 3 and Figures 2–5) indicated substantial acute load agreement with total draws (ICC = 0.6–0.8) and moderate agreement with training draws (ICC = 0.4–0.6); chronic load showed weak agreement with time-based metrics and total draws (ICC = 0.2–0.4) and similar weak agreement with training draws; ACWR demonstrated moderate agreement with time-based metrics and total draws but weak agreement with training draws; monotony exhibited weak agreement across all metrics; and pressure showed moderate consistency with time-based metrics and total draws but weak consistency with competition draws.

**Table 3:** Correlation analysis and consistency test table for external load and sRPE (*N* = 217).

Classification		Acute load		Chronic load		ACWR		Monotony		Training pressure		Curling load	
Curling duration	*r*	0.790**		0.508**		0.535**		−0.304**		0.379**		0.862**	
	ICC	0.816		0.464		0.621		−0.248		0.446		0.847	
	95% CI	0.741	0.840	0.562	0.353	0.697	0.532	−0.119	−0.368	0.503	0.278	0.881	0.805
Total duration	*r*	0.842**		0.524**		0.587**		−0.259**		0.459**		0.840**	
	ICC	0.847		0.469		0.677		−0.217		0.462		0.824	
	95% CI	0.805	0.881	0.566	0.358	0.743	0.597	0.087	−0.340	0.561	0.351	0.863	0.776
Training draws	*r*	0.473**		0.355**		0.252**		−0.184**		0.235**		0.472**	
	ICC	0.476		0.372		0.281		−0.218		0.213		0.460	
	95% CI	0.366	0.572	0.482	0.252	0.399	0.154	−0.047	−0.304	0.337	0.083	0.559	0.348
Competition draws	*r*	0.170*		−0.047		0.210**		0.122		0.253**		0.209**	
	ICC	0.064		−0.105		0.175		0.224		0.220		0.097	
	95% CI	0.196	−0.069	0.028	−0.235	0.301	0.043	0.347	0.094	0.343	0.090	0.227	−0.036
Total draws	*r*	0.544**		0.336**		0.343**		−0.106		0.373**		0.567**	
	ICC	0.603		0.438		0.453		−0.038		0.408		0.600	
	95% CI	0.673	0.500	0.460	0.226	0.553	0.341	0.096	−0.170	0.513	0.291	0.679	0.507

In correlation analysis: **Significantly correlated at the 0.01 level (bilateral); *Significantly correlated at the 0.05 level (two-sided).In the ICC intragroup correlation coefficients, <0.2 is poor correlation, 0.2–0.4 is fair correlation, 0.4–0.6 is moderate correlation, 0.6–0.8 is strong correlation, and 0.8–1.00 is very strong correlation.

#### sRPE and physiological and biochemical indicators

**Figure 6:**
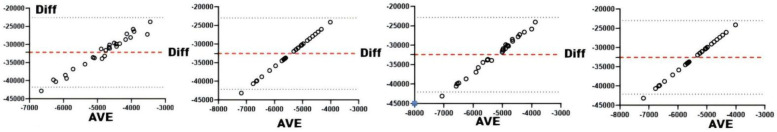
Bland-Altman plots of physiologic and biochemical indices and AL. From left to right: Bland-Altman plots of short-term loading and testosterone, cortisol, creatine kinase, and hematocrit.

**Figure 7:**
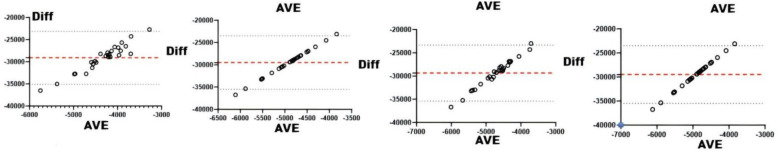
Bland-Altman plots of physiologic and biochemical indices and CL. From left to right: Bland-Altman plots of long-term loading and and testosterone, cortisol, creatine kinase, and hematocrit.

**Figure 8:**
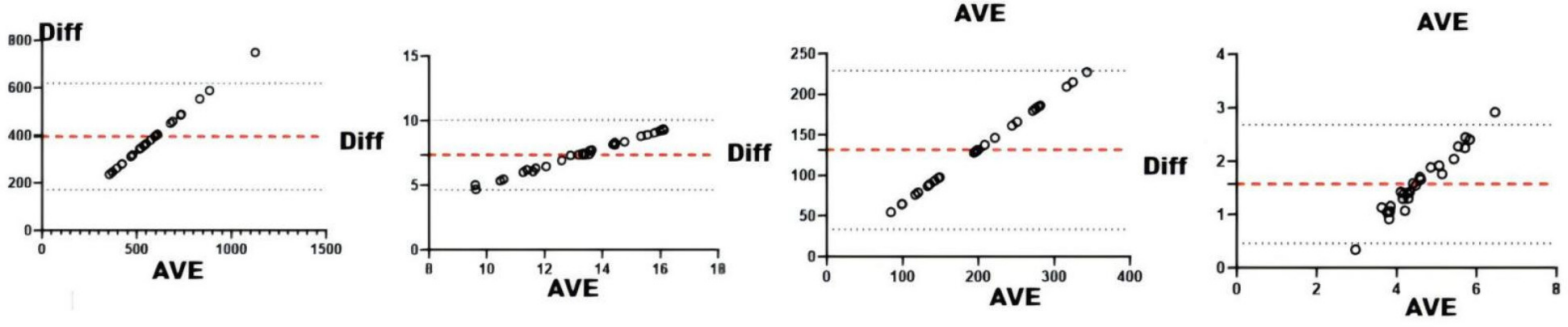
Bland-Altman plots of physiologic and biochemical indices and ACWR. From left to right: Bland-Altman plots of ACWR and testosterone, cortisol, creatine kinase, and hematocrit.

Correlation analyses between physiological/biochemical markers and sRPE (Table 4) revealed cortisol significantly correlated with ACWR (*r* < 0.05), while blood urea showed significant correlation with CL (*r* < 0.05); all other pairings were non-significant. Consistency testing (Table 4 and Figures 5–8) demonstrated: short-term load exhibited moderate agreement with blood urea (ICC = 0.40–0.59) but weak agreement with cortisol; long-term load showed moderate consistency with blood urea and weak consistency with creatine kinase; ACWR displayed weak agreement with testosterone and moderate agreement with cortisol.

**Table 4:** Correlation analysis and consistency test table of physiological and biochemical indicators and sRPE (*N* = 27).

Classification		Acute load		Chronic load		ACWR	
Testosterone	*r*	−0.084		0.048		−0.240	
	ICC	−0.086		−0.046		−0.241	
	95% CI	0.298	−0.445	0.413	−0.333	0.146	−0.564
Cortisol	*r*	−0.195		0.041		−0.400	
	ICC	−0.236		0.041		−0.421	
	95% CI	0.192	−0.531	0.409	−0.338	−0.030	−0.673
Creatine Kinase	*r*	−0.180		−0.249		−0.002	
	ICC	−0.180		−0.248		−0.002	
	95% CI	0.208	−0.519	0.139	−0.569	0.372	−0.376
Hematocrit	*r*	−0.362		−0.473		−0.073	
	ICC	−0.422		−0.474		−0.072	
	95% CI	0.013	−0.648	−0.122	−0.720	0.310	−0.435

In the ICC intragroup correlation coefficients, <0.2 is poor correlation, 0.2–0.4 is fair correlation, 0.4–0.6 is moderate correlation, 0.6–0.8 is strong correlation, and 0.8–1.00 is very strong correlation.

#### sRPE and omegawave competitive status evaluation system

**Figure 9:**
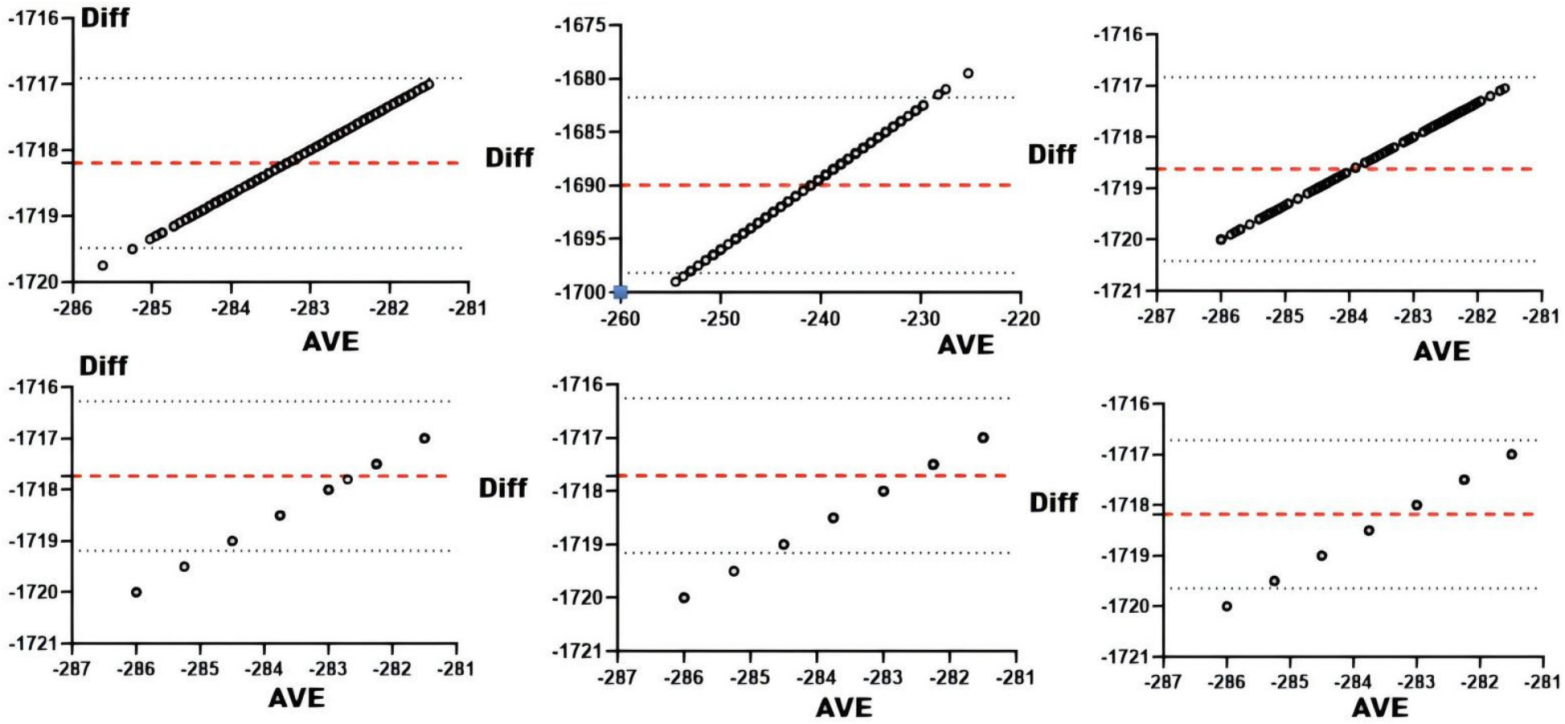
Bland-Altman plots of Omegawave athletic status evaluation indicators vs. daily training loads. From top to bottom and from left to right: Bland-Altman plots of daily training load vs. integrated readiness, resting heart rate, central nervous system functional status, cardiac functional status, cardiac regulatory system, and stress.

Correlation analyses between Omegawave indicators and sRPE (Table 5) demonstrated significant associations for daily training load with all parameters at *r* < 0.01. Consistency assessments (Table 5 and Figure 9) revealed daily load exhibited substantial agreement with integrated physiological state and cardiac function, while showing moderate agreement with resting heart rate, central nervous system status, cardiac regulation, and stress state.

**Table 5:** Table of correlation analysis and consistency test between Omegawave athletic Status evaluation indicators and sRPE (*N* = 275).

Classification		Daily load	
Comprehensive preparation	*r*	−0.576**	
	ICC	−0.679	
	95% CI	−0.600	−0.745
Resting heart rate	*r*	−0.347**	
	ICC	0.413	
	95% CI	0.500	0.274
Central nervous system	*r*	−0.443	
	ICC	−0.517	
	95% CI	−0.412	−0.608
Cardiac function system	*r*	−0.472	
	ICC	−0.608	
	95% CI	−0.517	−0.686
Cardiac regulatory system	*r*	−0.429	
	ICC	−0.565	
	95% CI	−0.467	−0.650
Pressure	*r*	−0.480	
	ICC	−0.518	
	95% CI	−0.413	−0.610

In correlation analysis: **Significantly correlated at the 0.01 level (bilateral). In the ICC intragroup correlation coefficients, <0.2 is poor correlation, 0.2–0.4 is fair correlation, 0.4–0.6 is moderate correlation, 0.6–0.8 is strong correlation, and 0.8–1.00 is very strong correlation.

## Discussion

### sRPE with external load

**Figure 10:**
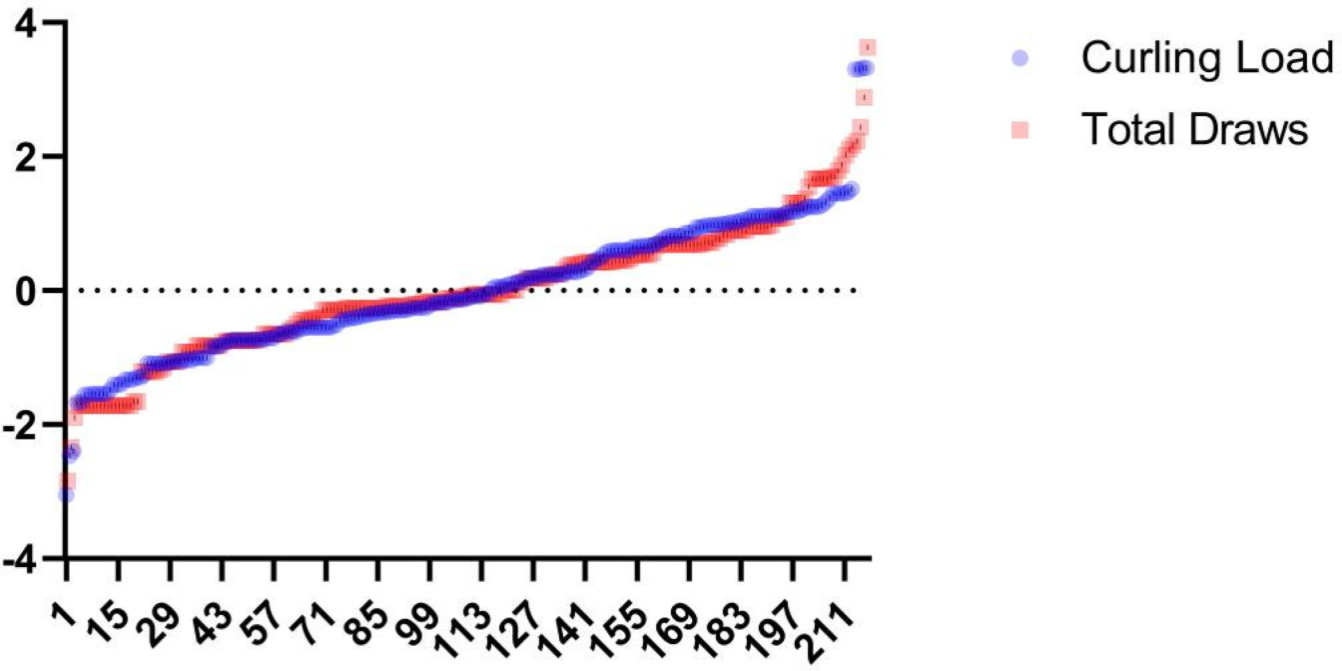
Specialized training load-total number of pitches curve. This graph is the result after the data has been standardized.

sRPE load metrics demonstrate strong correlation and consistency with most external load indicators, as evidenced by the high covariance with standardized training loads and total draws in Figure 10, affirming their utility for tracking external load variations. In curling, where physical exertion patterns remain relatively consistent across techniques and intensity primarily derives from ice sweeping and tactical cognition, the extended recovery periods during prolonged training/competition dilute acute physiological strain. Competition loads exhibit particular complexity due to: 1. strategic demands creating variable physical expenditure, 2. opponent strength disparities (intra-squad to international matches) causing mental exertion fluctuations—where superior opponents elevate sRPE through psychological stress while inferior opponents depress it through reduced engagement, and 3. the consistent phenomenon of lower draw volumes but higher sRPE values in matches vs. training, attributable to both heightened cognitive load and increased sweeping intensity from competitive mentality. Consequently, neither draw counts nor session duration—even in this cognition-dominated sport—adequately capture athletes' psychophysiological exertion, evidenced by significant intensity differences between equally timed training and competition. Furthermore, comparing sRPE against external loads reveals load sensitivity: divergent sRPE responses to statistically similar external loads may indicate high sensitivity (suggesting fatigue onset) or low sensitivity (indicating training adaptation), providing actionable biomarkers for athletic status that warrant further validation.

### sRPE and physiological and biochemical indicators

Cortisol and blood urea exhibited weak correlations with sRPE-quantified load, representing the only significant biochemical relationships. Consistency analyses revealed moderate agreement between both short- and long-term loads with blood urea, while ACWR showed moderate consistency with cortisol. Critically, cortisol demonstrated a negative correlation with ACWR, indicating that increased training volatility reduces cortisol concentration within physiological ranges. This suggests optimal load fluctuation mitigates chronic fatigue accumulation. Conversely, elevated long-term load correlated with increased blood urea, signifying physiological fatigue from excessive loading. These patterns collectively establish sRPE as a viable proxy for biochemical markers in load monitoring.

### sRPE and the omegawave athletic status evaluation system

**Figure 11:**
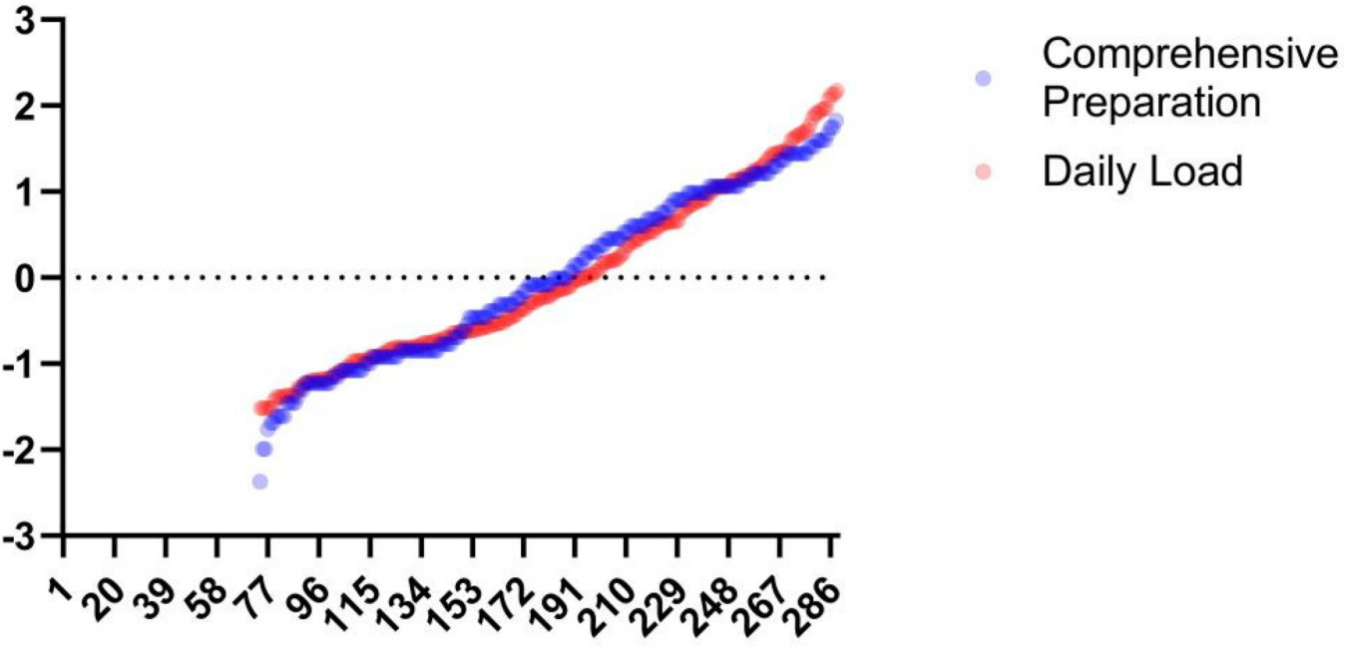
Daily load-integrated readiness curve. This figure shows the results after normalization of the data.

The Omegawave Athletic State evaluation and sRPE both assess athlete load states yet differ fundamentally (Figure 11). Omegawave precisely measures current physiological status but cannot isolate daily training load impact, as residual fatigue from prior sessions may elevate readings even during rest days. This temporal insensitivity limits its accuracy for single-session evaluation. While Omegawave testing surpasses biochemical markers in convenience, it remains more time-intensive than sRPE collection. Coaches requiring rapid daily load assessment should prioritize sRPE, whereas Omegawave better characterizes underlying physiological mechanisms. Crucially, sRPE reflects the organism's response to applied external load and effectively quantifies training impact when athletes reliably report subjective fatigue, though its subjectivity raises reliability concerns. External load metrics offer objective quantification with similar operational efficiency but fail to capture internal physiological strain or psychological exertion. Physiological biomarkers provide superior quantification precision yet require invasive procedures, strict collection protocols, and retrospective analysis—offering high-validity moment-state evaluation without predictive capacity. Omegawave's distinctive advantage lies in identifying directional load effects to guide training adaptations, though equipment dependency constrains practical implementation.

### Comparative analysis of bases

**Figure 12:**
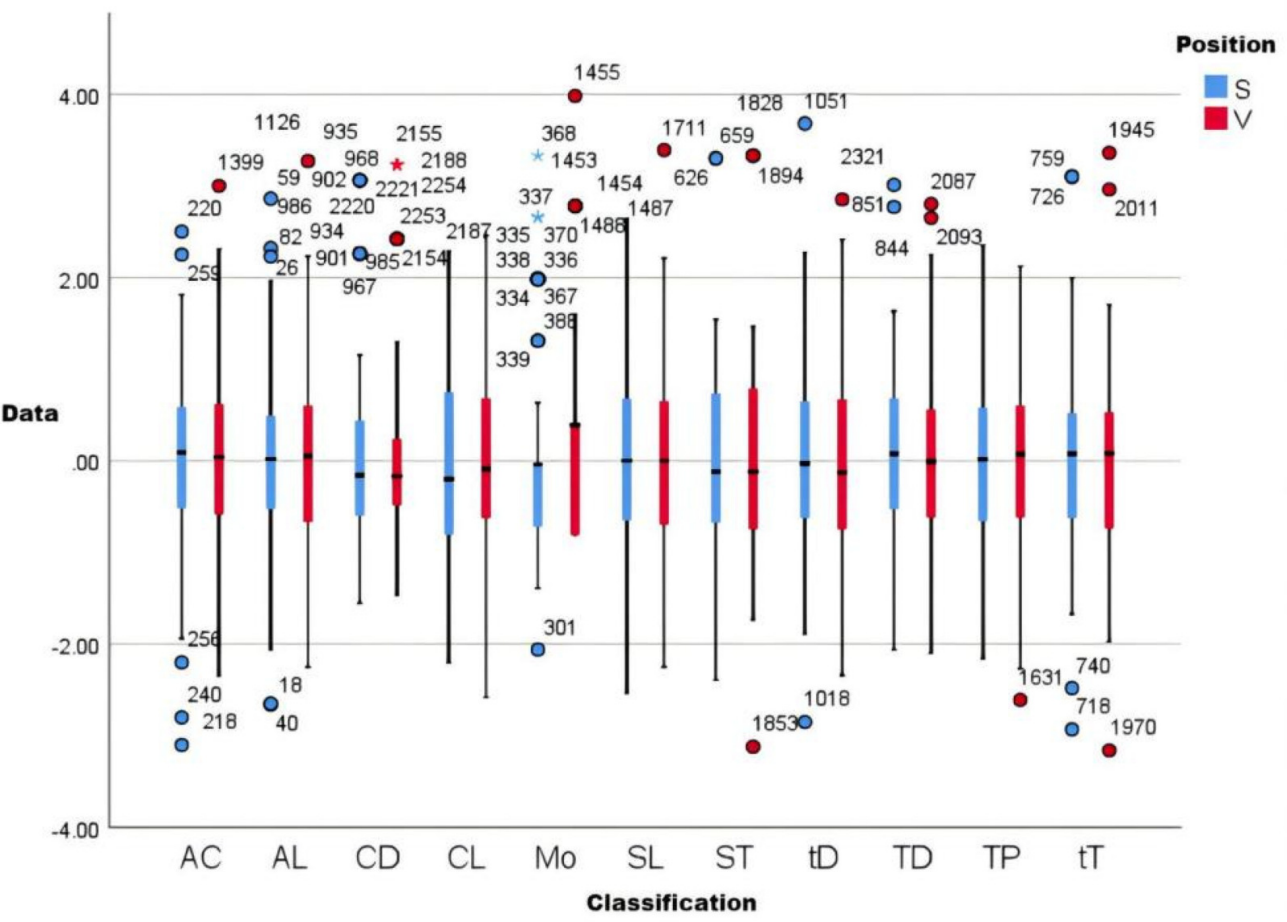
Comparative load analysis graph. This graph is the result of normalizing the data, where AC is acute chronic load ratio, AL is acute load, CL is chronic load, SL is specialized training load, Mo is monotonicity, TP is training pressure, TD is number of training draws, CD is competition draws, tD is total draws, ST is hours of curling, and sT is hours of all training. S is the ice sweeper (Position 1st and 2nd), V is the skip (Position 3rd and 4th).

In curling, front-end positions (S: first/second basemen) primarily execute ice sweeping while back-end positions (V: third/fourth basemen) direct tactical decision-making. Analysis of pre-competition cycle loads (Figure 12) revealed minimal differentiation between positions across external (draw counts) and internal (sRPE-derived) load metrics. Notably, significant training monotony divergence emerged, attributable to distinct load sources: S positions experienced predominantly physiological stress from sweeping, whereas V positions incurred cognitive demands influenced by shot difficulty and opponent tactics, thereby generating position-specific monotonicity profiles.

### Comparative analysis between four load quantification tools

sRPE provides superior assessment of the organism's response to external load when athletes accurately self-report, offering reasonable evaluation of training impact despite reliability concerns stemming from its inherent subjectivity. External load metrics deliver objective quantification with comparable operational efficiency but fail to capture internal physiological strain or psychological exertion. Physiological biomarkers afford greater quantification precision yet incur substantial time costs, require stringent collection protocols that limit utility to retrospective analysis, involve invasive procedures, and lack predictive capacity despite high validity for momentary state assessment. The Omegawave system's principal advantage lies in identifying directional load effects to inform training adaptations, though practical implementation faces portability constraints.

### Reflections on the degree of load quantification

Different training load quantification methods exhibit distinct characteristics, ranging from exceptionally precise quantitative tools (accurate to 2–3 decimal places) to qualitatively analytical approaches. When selecting appropriate quantification methods, coaches and researchers must consider not only sport-specific requirements, athlete proficiency levels, and training phase demands, but also required precision thresholds. In practical training contexts, single-decimal accuracy typically suffices for fatigue assessment relevance, making precision needs a critical selection criterion. Given varying theoretical and applied values across quantification tools, deliberate evaluation of methodological alignment with both research objectives and practical utility remains essential.

### Practical applications

sRPE constitutes a reliable indicator for evaluating training load in curling programs. The sRPE-workload assessment process demonstrates operational convenience, facilitates phased evaluation of training loads, and serves as an effective tool for coaches and multidisciplinary support teams to implement load management strategies. The sRPE evaluation methodology exhibits inherent limitations, including the absence of categorical differentiation of load magnitudes, susceptibility to subjective influences, and potential cumulative overestimation of training loads when applied across extended temporal frameworks. Prudent selection of heterogeneous load assessment methodologies should be predicated on the practical demands of training programs.

## Conclusion

The session-Rating of Perceived Exertion (sRPE) demonstrates reliable curling training load monitoring through operational simplicity and phased assessment capabilities, proving valuable for coaching load management. However, methodological limitations persist, including unclassified load weighting, subjective bias susceptibility, and potential load-stacking artifacts during extended monitoring. While algorithmic refinements have been proposed, they typically undermine sRPE's inherent practicality. Our findings consequently advocate context-specific selection of load quantification tools aligned with distinct training objectives.

## Limitation

This study was conducted outside strictly controlled laboratory conditions within an actual Olympic preparation context. While inherent constraints in experimental design, including limited physiological biomarker sampling, may introduce bias, the research retains significant practical validity. Furthermore, potential time-lag effects in physiological and biochemical indicators may particularly complicate longitudinal analyses.

## Data Availability

The original contributions presented in the study are included in the article/Supplementary Material, further inquiries can be directed to the corresponding author.
